# Aluminum Matrix Composite (AlSi7Mg2Sr0.03/SiC_p_) Pistons Obtained by Mechanical Mixing Method

**DOI:** 10.3390/ma11010042

**Published:** 2017-12-28

**Authors:** Maciej Dyzia

**Affiliations:** Faculty of Materials Engineering and Metallurgy, Silesian University of Technology, Krasińskiego 8, PL 40-019 Katowice, Poland; maciej.dyzia@polsl.pl; Tel.: +48-32-603-4368

**Keywords:** metal-matrix composites (MMCs), casting, machining, wear properties

## Abstract

Metal matrix composites are undoubtedly a group of advanced engineering materials. Compared to unreinforced matrix material, they are characterized by increased strength, greater stiffness, increased wear resistance, better mechanical properties and dimensional stability at elevated temperatures as well as lower density. Due to its very favorable tribological properties for many years research has been conducted on the application of MMC in friction node. The article presents important technological aspects related to the production and properties of composite pistons. Under industrial conditions, a composite suspension (AlSi7Mg2Sr0.03/SiC_p_ 10 vol %) was prepared to allow casting of the semi-finished pistons series. Machining parameters of the working surfaces of the piston were selected on the basis of the turning test made on PCD, PCNM and uncoated carbide tools. The tribological properties of the composite pistons were determined on the basis of the pin-on-disc and the abrasion wear. The scuffing tests carried out under real operating conditions have confirmed the possibility of using composite pistons in air compressors.

## 1. Introduction

Metal matrix composites (MMC) are a wide range of materials in which aluminum, beryllium, magnesium, titanium, iron, nickel, cobalt and silver alloys can be used as a matrix. Ceramics such as SiC, Al_2_O_3_, B_4_C, TiC, TiB, graphite, carbon fibers and tungsten or steel fibers can be applied as materials for reinforcing. The reinforcing phases can take the form of dispersive particles formed with in situ reactions, particles, short fibers or wiskers, continues fibers or monofilaments and porous preforms. In general, metal matrix composite manufacturing methods can be divided into: (1) liquid-state processing; (2) solid-state processing; (3) vapour-state processing [1]. The strong interest in the composite materials are result from a number of their properties, which can be designed by the proper selection of reinforcing components (volume fraction, shape and size), chemical composition of matrix and technological parameters.

According to many authors [2,3,4,5,6], the potential areas of MMC applications are: (1) parts for ground vehicles, such as brake rotors and discs, pistons, liners and connection rods; (2) parts for military and civil aircrafts, such as axle tubes, blade and gear box casing, fan and compressor blades, turbine blades; (3) for electronic packaging and thermal management, such as microwave packaging and microprocessor lids.

Among MMC materials, the composites with aluminum alloy matrix (AlMCs) are the largest group, mainly due to their favourable properties such as low density (2.6–3.2 g/cm^3^), thermal conductivity (120–177 W/m K), specific strength (70–250 kNm/kg), wear resistance (0.02–0.43 mm^3^, ASTM G-77) and the ability to be formed and treated on conventional equipment [3,7,8]. With these properties aluminum matrix composites can replace monolithic materials such aluminum, ferrous, titanium alloys and polymer-based composites in several applications [3,6]. AlMCs are mainly produced by powder metallurgy processes (solid-state) and melting metallurgy processes (liquid-state) [3,9,10]. In liquid-state processes, the phenomenon of wetting of solid ceramics by liquid metal plays an important role. The introduction of ceramic particles to the matrix can take place: during mechanical mixing of alloy, addition of composite briquettes in the melt followed by mild mixing, injection in the melt with an inert gas, addition to ultrasonically irradiated or electromagnetically stirred melt and centrifugal dispersion of particles. While for molding in foundry processes the following are used: gravity sand or die casting, low pressure casting, high pressure die casting, squeeze casting, centrifugal casting, vacuum casting and investment casting. In the case of infiltration of porous preforms mainly the pressure methods are used [4,11,12,13,14].

Due to the low density and wear resistance of AlMCs the automotive industry is indicated as the main field of its application [5]. Well known examples of applications are: Toyota diesel engine pistons containing Saffil (Al_2_O_3_) short fiber insert and hybrid material made of short alumina and carbon fibers infiltrated with molten aluminum alloy in the Honda engine block [3]. Prasad and Asthana [15] presented more proven applications of cast AlMCs in the automotive industry. The properties of Al alloys and AlMCs for automotive application are presented in Table 1.

A significant group in these applications are AlMCs reinforced with SiC or Al_2_O_3_ particles obtained by melting metallurgy processes. Under industrial conditions, composite elements are most often produced by squeeze casting or infiltration methods. Due to the cost of launching the production, it is profitable for large series. In the case of short-run production, the mechanical mixing (stir-casting) method is more profitable [16,17].

This article summarized the results of research work on production of composite by mechanical mixing method, which allowed development of composite pistons for air compressors. The important factors determining the properties of the composite obtained by mechanical mixing include: preparation of the matrix alloy (refinement, alloy composition modifications), preparation of ceramic particles (chemical preparation, thermal processing), as well as the speed and duration of the suspension mixing process. The conditions of homogenization are crucial for the interface and distribution of ceramic particles in liquid metal [18,19,20,21,22]. Distribution of reinforcement in turn influences the structure of the composite material formed by casting. However, the structure and especially the interface between matrix alloy and reinforcement have significant influences on composite properties [23,24]. A number of studies have shown that the interface bond between particle and matrix is the one of the reason of composite wear property [25,26,27].

## 2. Material and Methods

As in other liquid-state processes, the phenomena that determine the permanent matrix bonding and reinforcement in the mechanical mixing method are wetting and reactivity in the molten metal and ceramic reinforcing phase. In the aluminum alloy-SiC system, the modification of alloy composition limits the reaction (formation of Al_4_C_3_) and improves the wettability in the system [13,28,29,30].

**Figure 1:**
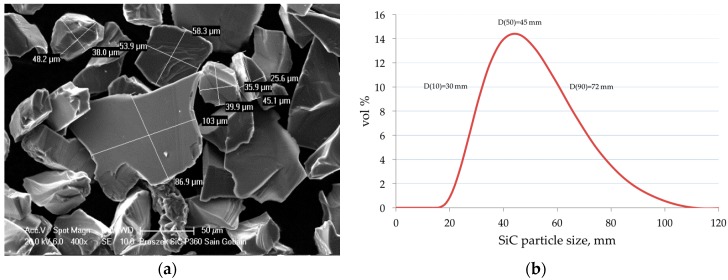
(**a**) SEM image of SiC reinforcement particles; (**b**) particle size distribution.

As the matrix material, the commercially available cast aluminum alloy EN AC 42200 (AlSi7Mg0.6) was applied. As the reinforcement the black silicon carbides grains Saint-Gobain SIKA^®^ ABR P360 (Saint-Gobain Ceramic Materials, La Defense CEDEX, France) (avg. particle size 45 μm, Figure 1) was used. In order to improve the wetting conditions in the ceramic-metal system, the composition of the matrix alloy was modified by the addition of Mg and Sr. As the master alloys, AlMg25 and AlSr10 produced by the Institute of Non-Ferrous Metals, Light Metals Division (Skawina, Poland) were used. On the basis of the results of previous studies [21,28], taking into account the production costs and properties of composite materials [31], 10 vol % fraction of SiC reinforcement was selected to produce of composite pistons.

Composite suspension (AlSi7Mg2Sr0.03/SiC_p_ 10 vol %.) under semi-technical conditions was made in the autoclave furnace PTA 200/PrG (Czylok, Jastrzębie, Poland) according to the procedure described in the papers [20,21,22]. The following process parameters were set: (1) melting of AlSi7Mg0.6 alloy at 720 °C and one hour refining with 5 L/min flow of argon 4.9 (Messer Polska, Chorzów, Poland) with a graphite stirrer (own constructed, SUT, Katowice Poland); (2) modification of chemical composition of base alloy to by adding 2 wt % of Mg and 0.03 wt % of Sr, after the introduction of master alloys the modified matrix alloy was mixed under reduced pressure conditions (50 hPa) at 720 °C for 1 h; (3) introduction of reinforcement particles onto the vortex surface of metal in the amount of 200 g/min, SiC particles were earlier preheated in air at 700 °C for 24 h and then directly before the introduction at 350 °C for 1 h; (4) two hours homogenization and degassing of the suspension of AlSi7Mg2Sr0.03/SiC_p_ 10 vol % under reduced pressure conditions (50 hPa) at 720 °C.

At each stage of the suspension production, test castings were taken into standardized sensor sand mold QC4080 (Heraeus Electro-Nite Polska, Sosnowiec, Poland) with K-type thermocouple for registration of changes in temperature during the cooling phase. The solidification curves were recorded for the base AlSi7Mg0.6 alloy after melting and after refining, after modification and composite suspension after the homogenization stage and after the last casting. Polished microsection from test ingots after melting and after refining were applied to assign the hydrogen content. The investigations were carried out using LECO RH EN-602 hydrogen analyzer (LECO Corporation, Saint Joseph, MI, USA) according to the measurement procedure at the Institute of Non-Ferrous Metals, Light Metals Division.

**Figure 2:**
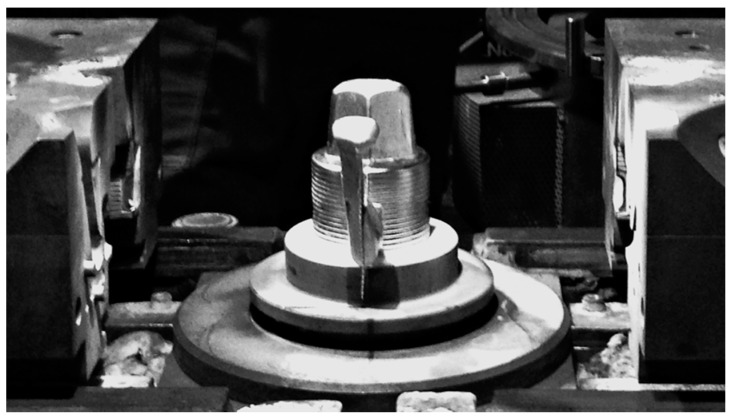
Mold cast of semi-finished AlSi7Mg2Sr0.03/SiC_p_ 10 vol % composite piston.

The composite suspension AlSi7Mg2Sr0.03/SiC_p_ 10 vol % was cast at 720 °C and the average time of casting per piston was approximately 130 s. 85 semi-finished pistons were cast under the industrial conditions according to the manufacturer’s procedure (Złotecki Ltd., Rojewo, Poland) (Figure 2).

**Figure 3:**
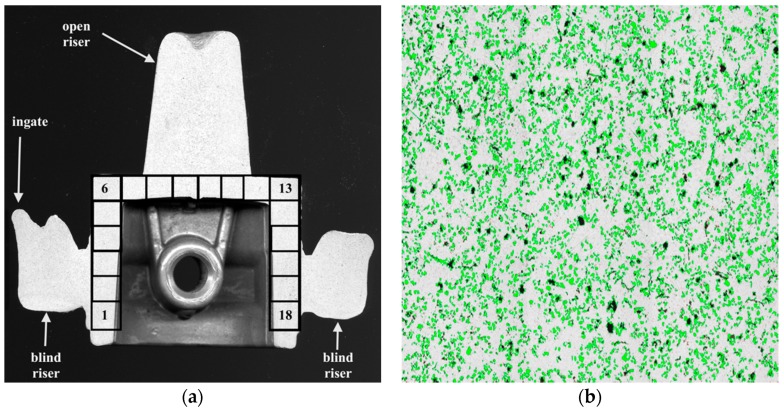
Particle distribution within the cast: (**a**) cross section of composite piston with marked 18 measurement areas marked; (**b**) sample of single analyse image, surface area 4.58 mm^2^.

According to the plan of previous experiments, the semi-finished pistons no. 1 and 30 of the casting series were intended to evaluate the reinforcing particles distribution within the cast [32]. The distribution assessment was made with 18 measurement areas of 41.3 mm^2^ (composed of 3 × 3 images at 50× magnification, Figure 3). To acquire the images of microstructure Olympus GX71 light microscope (Olympus Corporation, Tokyo, Japan) was used. These studies were carried out at the Institute of Non-Ferrous Metals, Light Metals Division [33].

Out of the others semi-finished pistons produced, 20 pistons were intended for the final processing at the Złotecki company and the remaining ones were used for the selection of machining parameters at the Institute of Advanced Manufacturing Technology (Cracow, Poland). The turning tests were carried out with the machining centre NL2000 SY (DMG Mori Seiki Co., Nagoya, Japan). The machining parameters were based on the limited service life of the turning tool. The criteria for assessing the use of the tool were presented in [22]. The images of the turning tools after composite machining were taken with the JEOL JSM 6460LV scanning microscope.

From open riser parts (Figure 3a) adjacent to the piston head samples were taken for the wear tests. The tribological tests using the versatile mechanical tester CETR UMT-2M (Center for Tribology Inc. Bruker Nano Inc., Campbell, CA, USA) in the pin-on-disc configuration were carried out at the Institute of Advanced Manufacturing Technology and the abrasion wear tests using the Taber Rotary Abraser tester were made at the Light Metals Division of the Institute of Non-Ferrous Metals [34]. The Brinell hardness measurements were also taken on the surface of the piston crown by Duramin 2500E tester (EMCO-TEST Prüfmaschinen GmbH, Kuchl, Austria) with 2.5 mm ball intruder and 613 N load force. The piston scuffing tests under the real conditions of air compressor were carried out on the special test stand at the compressor manufacturer FOS Polmo JSC (Łódź, Poland).

## 3. Results and Discussion

### 3.1. Refining and Modification of Matrix Alloy

**Figure 4:**
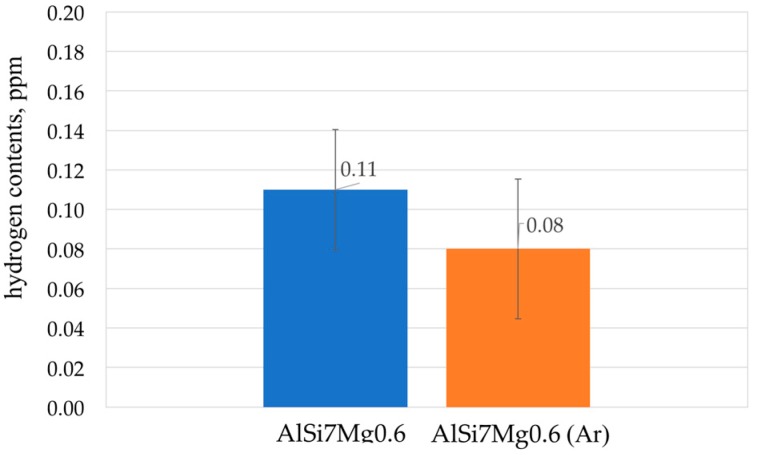
Effect of refining base AlSi7Mg0.6 by Ar barbotage 5 L/min.

**Figure 5:**
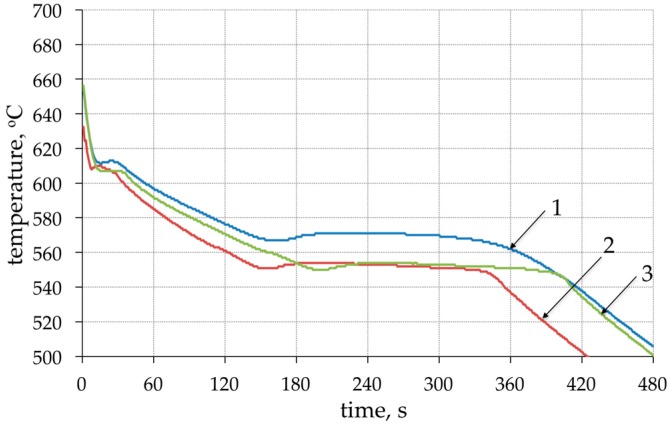
Solidification curves: 1—base AlSi7Mg0.6 alloy; 2—AlSi7Mg0.6 after Ar flow refining; 3—AlSi7Mg0.6 after addition 2 wt % Mg and 0.03 wt % Sr.

**Figure 6:**
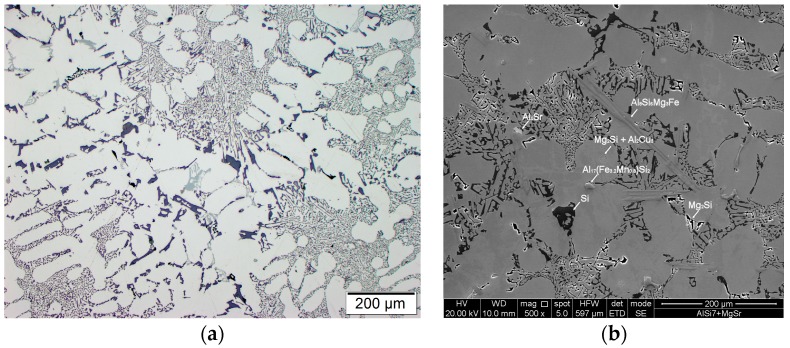
Structure of AlSi7Mg0.6 after addition 2 wt % Mg and 0.03 wt % Sr, (**a**) LM; (**b**) SEM.

Modification of chemical composition of the cast aluminum alloys is commonly used to improve the mechanical properties. The additions of modifiers such as Sr, Na, Ca, Ba or Eu modify the eutectic-Si morphology and have a beneficial effect on both strength and ductility [35,36,37,38,39,40,41]. In the presented method for producing the composite suspension, refining with Ar by barbotage and modification with magnesium and strontium plays an important role in liquid metal properties. The refining removes non-metallic inclusions and reduces hydrogen content in AlSi7Mg0.6 (Figure 4). While the addition of Mg and Sr reduces surface tension and limits the formation of an amorphous Al_2_O_3_ film, which improves the wettability of the ceramic metal system [38,39,40]. The effect of the refining and modification of liquid AlSi7Mg0.6 is also visible in the solidification curves (Figure 5). The decrease in solidification temperature to 554 °C after modification of AlSi7Mg0.6 base alloy by addition of 2 wt % Mg and 0.03 wt % Sr increases the degree of matrix overheating during suspension production. The structure of modified matrix alloy (AlSi7Mg2Sr003) is shown in Figure 6.

A more complete description of the interaction of modifiers with aluminum matrix and reinforcement ceramic phases is presented in [41,42].

### 3.2. Composite Suspension Stability

**Figure 7:**
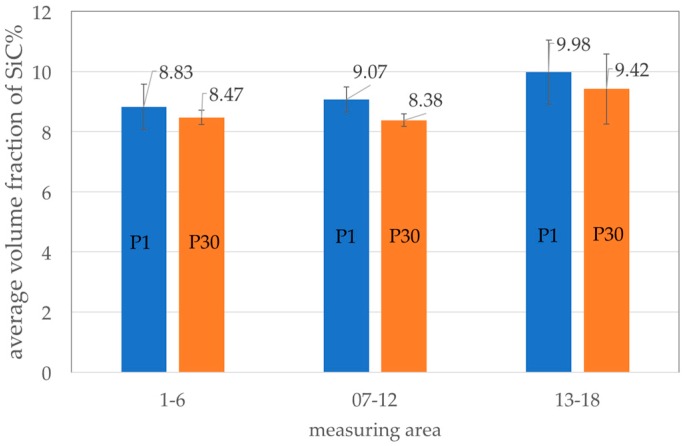
Distribution of the SiC particles in the semi-finished piston: P1—1st cast, P30—30th cast.

An important step in the production of composite suspension is homogenization to provide a uniform distribution of particles over the entire volume of liquid metal. Stability of the AlSi7Mg2Sr0.03/SiC_p_ 10 vol % composite suspension was assessed on the basis of SiC particle distribution in the semi-finished piston in the three areas marked in Figure 3a: at the ingate site (area 1–6), in the piston crown (area 7–12) and at the opposite side to the ingate (area 13–18). The average volume fraction of SiC particles in these areas is shown in Figure 7.

**Figure 8:**
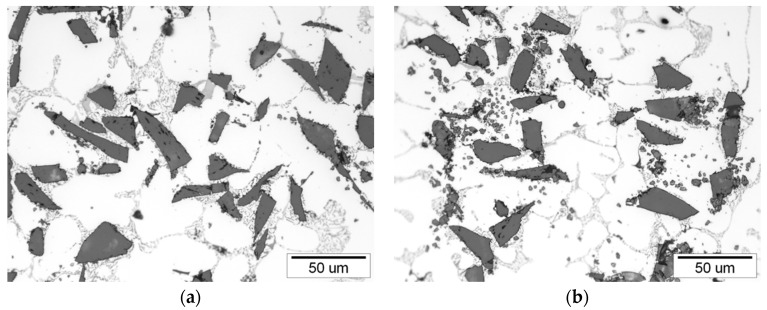
Microstructure of cast AlSi7Mg2Sr0.03/SiC_p_ 10 vol % composite: (**a**) 1st semi-finished piston; (**b**) 30th semi-finished piston.

There was a tendency for the increase in volume fraction of reinforcement with a distance from ingate. For the first cast piston (P1) the difference between areas (1–6) and (13–18) was 1.15%. Similarly, for the 30th cast piston (P30) the difference between the areas (1–6) and (1–18) was 0.95%. However, taking into account the accuracy of the measurement (between 0.41% and 1.06%) it can be considered that the distribution of SiC particles in the cast pistons is uniforms. Therefore, it can be assumed that the composite suspension is stable during casting of the 30-piston series. The microstructure of the first and the last AlSi7Mg2Sr0.03/SiC_p_ 10 vol % composite cast in the series is shown in Figure 8.

**Figure 9:**
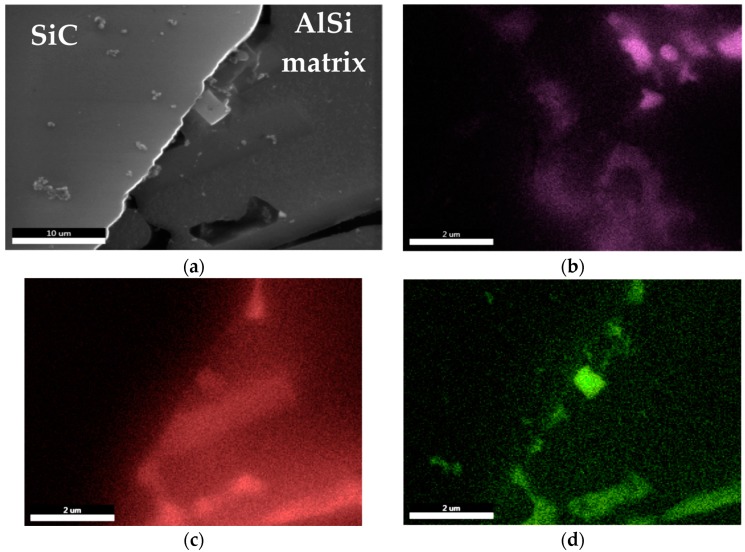
Surface distributions of elements at the interface between AlSi7Mg2Sr0.03 matrix alloy and SiC particles: (**a**) SEM image; (**b**) Sr K; (**c**) Mg K; (**d**) O K.

The stability of the suspension is also influenced by the interface between the SiC particles and the matrix alloy. As shown by the studies on distribution of elements around ceramic particles, the Mg-O-Sr system phases are formed (Figure 9). The presence of these phases limits the reactivity in the Al-SiC system and promotes the stability of bonding between the matrix and the reinforcement particles.

### 3.3. Machining Parameters of Composite Piston Skirt

**Figure 10:**
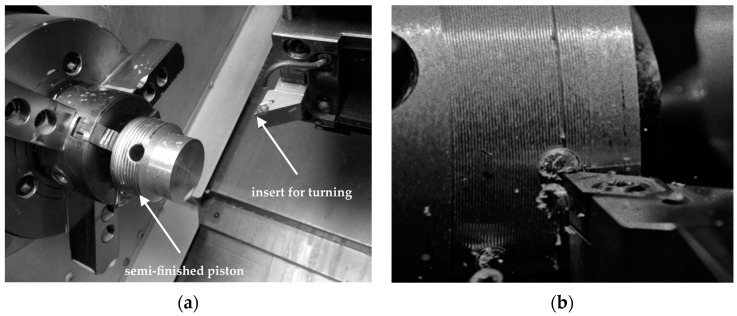
Touring tool life tests on piston, (**a**) tool holder; (**b**) machined surface.

After cutting off the gating system, the semi-finished pistons were subjected to the machining tests (Figure 10). The commercially available turning tools with polycrystalline diamond (PCD), polycrystalline cubic boron nitride (PCBN) and uncoated carbide (H10) inserts were used in the tests. All PCD and PCBN inserts were soldered on a sintered carbide substrate in one corner of the plate. The inserts marked MD220 (Mitsubishi Materials Corp., Tokyo, Japan) were made on PCD powder and designed to machining aluminum alloys with high cutting speeds. The inserts were made on: sintered cBN, TiC and Al_2_O_3_ powders (Mitsubishi MB710, Tokyo, Japan), cBN, TiN and Al_2_O_3_ sintered with particle activation (Mitsubishi MB8025, Tokyo, Japan), high content of cBN on special bonding matrix (Mitsubishi MB4020, Tokyo, Japan). All PCBN inserts are designed for universal machining applications. The H10 (AB Sandvik Coromant, Sandviken, Sweden) inserts are used for rough to finish turning of aluminum alloys. For testing, the turning tools with PCD inserts made at the Institute of Advanced Manufacturing Technology (Cracow, Poland) were also used. The inserts were produced by high pressure—high temperature sintering with 3–6 μm diamond powder in the TiB_2_ + Co matrix [43]. The results of turning tests are presented in Table 2.

**Figure 11:**
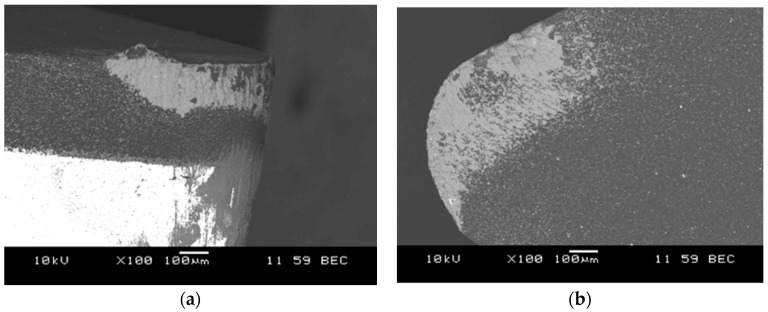
SEM image of DCMW PCD cutting edge after composite machining, wear by accumulations: (**a**) side view; (**b**) top view.

**Figure 12:**
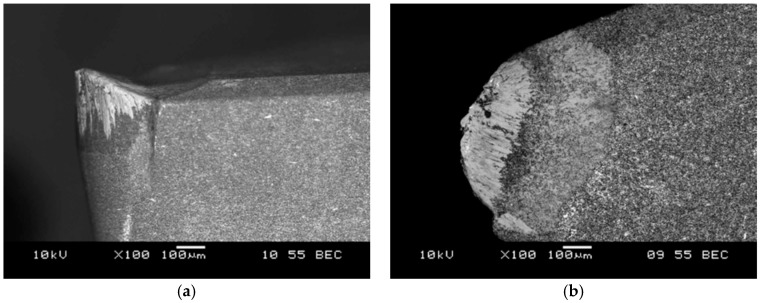
SEM image of DCGW PCBN cutting edge after composite machining, wear by accumulations: (**a**) side view; (**b**) top view.

**Figure 13:**
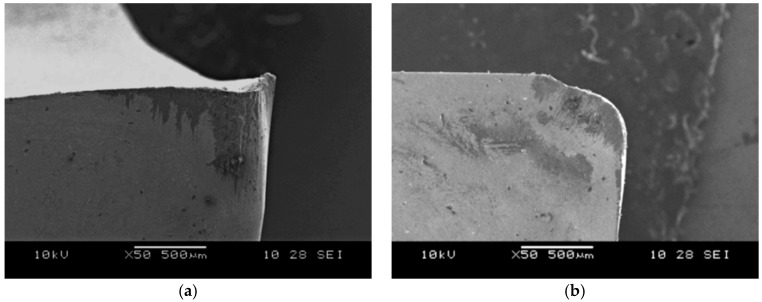
SEM image of CCGX H10 cutting edge after composite machining, wear by attrition: (**a**) side view; (**b**) top view.

These studies have confirmed hard machinability of aluminum matrix composites. The tool life was influenced primarily by the cutting speed. The obtained results also confirmed that the machined surface roughness of the treated surface (Ra), especially at the small radius of the side cutting edge angle (*r* = 0.4 mm) was mostly influenced by the feed rate. With a reduction in cutting speed to 300 m/min at feed rate of 0.10 mm/rev and 0.5 mm cutting depth, the tool life was increased approximately to 15 min. Hard machinability of the AlSi7Mg2Sr0.03/SiC_p_ 10 vol % composite material was also demonstrated by the results obtained at cutting speeds of 200 m/min, feed rate of 0.15 mm/rev and cutting depth 1.5 mm. The life of the PCD blade in cutting process was 12.3 min, which is 2.46 km per cutting length. According to the manufacturer's data, the amount of polycrystalline diamond used during the treatment of aluminum alloy with high Si content (without reinforced SiC phase) at 200 m/min, feed rate of 0.15 mm/rev and cutting depth of 1.5 mm exceeds 24 km. The images of the worn cutting edge after machining AlSi7Mg2Sr0.03/SiC_p_ 10 vol % composite are shown in the Figure 11, Figure 12 and Figure 13.

**Figure 14:**
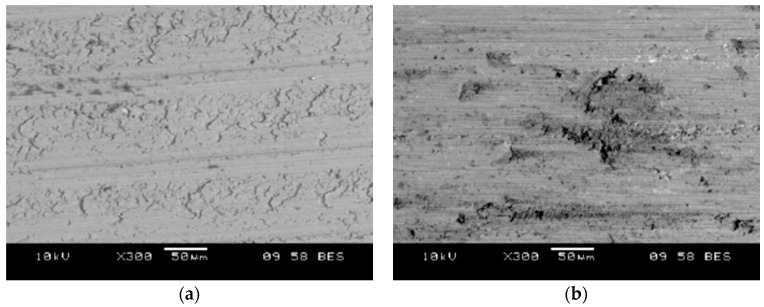
SEM image of surface after machining with DCMW PCD tool: (**a**) AlSi7Mg2Sr003 base alloy; (**b**) AlSi7Mg2Sr0.03/SiC_p_ 10 vol % composite.

The comparison of the surface of turned pistons is shown in (Figure 14). The analysis of the relief left by the cutting tool shows that the material was treated by picking and deforming, resulting in the appearance of both the scratches and the characteristic accumulations. The passage of the tool through the SiC causes it to be cut. Some parts of the particles remain in the matrix, while the others are crushed and pressed into the matrix material. No tearing off of SiC particles was observed on the machined surface.

On the basis of the obtained results, the parameters for machining the working surfaces of pistons under industrial conditions were selected. The stages of the piston surface forming process are shown in the paper [22].

### 3.4. Tribological Properties

**Figure 15:**
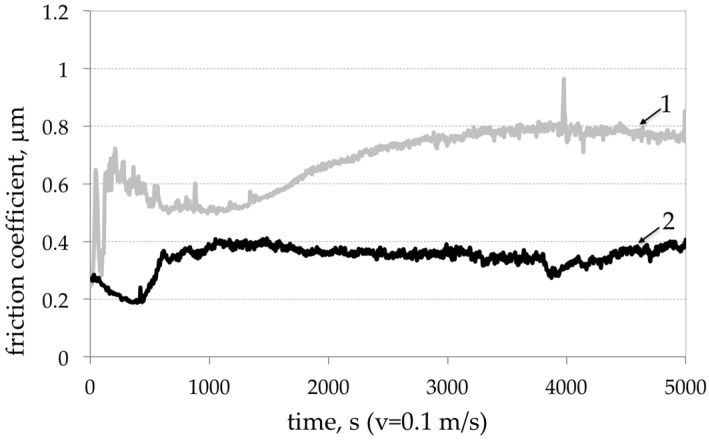
Comparison of friction coefficient changes in time: 1—AlSi7Mg2Sr003 base alloy; 2—AlSi7Mg2Sr0.03/SiC_p_ 10 vol % composite.

The laboratory tests under the pin-on-disc and abrasion conditions were used to evaluate the tribological properties of the composites. In the pin-on-disc configuration, the test sample was a cast iron pin with diameter of 5 mm, loaded with 2 N. The counter-test sample was AlSi7Mg2Sr0.03/SiC_p_ 10 vol % composite discs. The tests were carried out with friction radius of 10 mm and friction linear speed of 6000 mm/min at a distance of 500 m. As it can be seen in Figure 15 under the technically dry friction condition of the pin on disk system, the friction coefficient of the AlSi7Mg2Sr0.03/SiC_p_ 10 vol % composite after the initial grinding has stabilized in the range of 0.36–0.39. For the AlSi7Mg2Sr003 matrix alloy after grinding period, the friction coefficient increases from 0.5 to 0.8.

**Figure 16:**
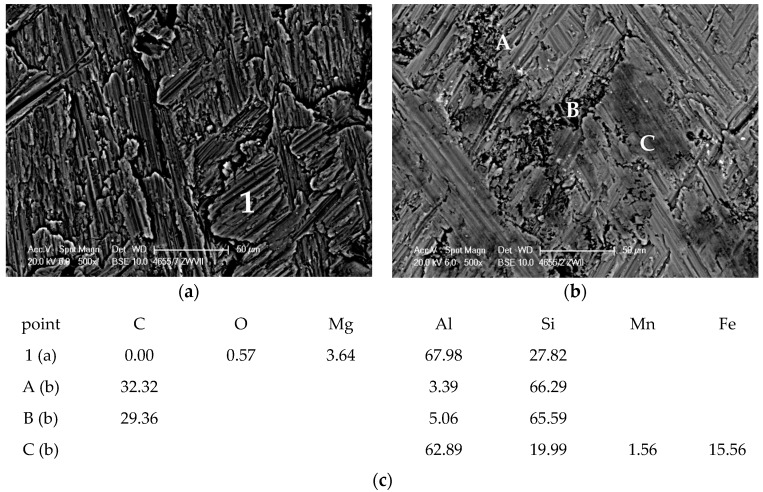
SEM image of worn out surface after abrasion test (**a**) AlSi7Mg2Sr0.03 base alloy; (**b**) AlSi7Mg2Sr0.03/SiC_p_ 10 vol % composite; (**c**) EDS analysis of elements at points.

The results obtained in the Taber Rotary Abraser test also confirm the favorable effect of the SiC reinforcing phase on tribological properties of the composite material. The analysis of worn out surface indicates that, as in the case of machining, the SiC particles are crushed and pressed into the matrix. Crushed particles strengthen the matrix alloy and limit its wear. As indicated by the analysis of the surface distribution of elements, the abrasion wheels disk wear products are also deposited on the worn surface of composite sample (Figure 16b—area in point C). The results of mass lost during the Taber Rotary Abraser test and the Brinell hardness tests are presented in Table 3.

**Figure 17:**
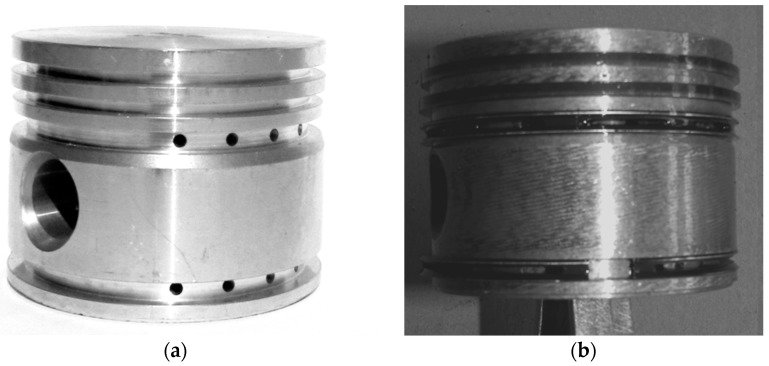
AlSi7Mg2Sr0.03/SiC_p_ 10 vol % composite piston of air compressor: (**a**) after machining; (**b**) after scuffing test.

The next test was of great significance for the evaluation of the properties of AlSi7Mg2Sr0.03/SiC_p_ 10 vol % composites under the real work conditions. From the series of ready-made composite pistons (Figure 17a), eight ones were selected and installed in air compressors made by FOS Polmo JSC (Łódź, Poland).

The scuffing test at air the compressor was started with 2000 rpm. By controlling air pressure in the tank and increasing the rotational speed a constant compressed air temperature of 200 °C was obtained. The compressor was cooled by 4 m/s air flow and with 2340–2490 rpm and the compressed air of 550–560 kPa pressure reached the temperature of the 200 °C. The compressor worked under these conditions for 15 min. Then the speed and cooling rate were being changed to raise the temperature at 10 °C intervals up to 290 °C. The compressor worked for 15 min at each temperature level. The compressor reached the temperature of 300 °C at 2980–3200 rpm. Under these conditions the compressor worked for one hour without cooling. After testing the compressor was disassembled and the pistons and cylinders were inspected visually. All AlSi7Mg2Sr0.03/SiC_p_ 10 vol % composite pistons have successfully passed the scuffing test, which is performed during the inspection of the mass-produced air compressors. The test results indicate that the pistons can be used in air compressors.

## 4. Conclusions

The tests carried out under the industrial conditions have confirmed that the adopted procedure for the production of aluminum matrix composite is correct. Modification of chemical composition of matrix alloy by addition of 2 wt % Mg and 0.03 wt % Sr and the process of homogenization of composite suspension are favorable for the reinforcement and matrix bonding. The stability of composite suspension enabled casting of a series of semi-products where distribution of reinforcing SiC (10 vol %) particles were uniform. It can therefore be assumed that this method makes it possible to produce short batches of composite castings with a total mass of approximately 50 kg.

The selection of the cutting process parameters allowed forming the working surfaces of the pistons. The composite pistons have passed the dimensional control and scuffing test under real operating conditions in air compressors. The positive result of the scuffing tests allows the composite pistons to be used in place of non-reinforced pistons.

The presence of 10 vol % SiC (45 μm) particles in the AlSi7Mg2Sr0.03 matrix increases the hardness of the piston crown, stabilizes the friction coefficient under the technically dry friction conditions and reduces the wear in the abrasive condition.

It should be noted, however, that the production of a composite piston is more expensive, especially the cost of machining is considerably higher. Therefore, further research is aimed at demonstrating the benefits of using composite pistons, especially in terms of improving the performance of the air compressor.

## Figures and Tables

**Table 1:** Properties of Al alloys and AlMCs for automotive application [3].

Material	Stiffness, GPa	YS, MPa	UTS, MPa	Elongation, %	CTE (10^−6^ K^−1^)
AlCu4Mg1	73	140–400	180–460	2–15	24.7
AlSi5A	70	90	100–140	10–15	23.7
AlMg1SiCu	70	85–240	120–300	3–18	23.0
AlSi7Mg0.3—T6	72	190	234	2–3.5	23.2
AlSi9Cu3(Fe)	73	140	160–240	1–3	22.3
AlSi9Mg0.3/SiC_p_ 20 vol %—T6, stir casting	98.6	338	359	0.4	17.5–21.4
AlCu4Mg1/SiC_p_ 25 vol %—T4, extruded	115	487	690	5	15.5
AlMg1SiCu/Al_2_O_3_ 15 vol %—T6, extruded	88.9	324	365		19.6–20.3
AlSi12CuMgNi/Safil short fiber 20 vol %, squeeze casting	105		310	0.2–0.5	app. 16

**Table 2:** Turning tool life tests parameters.

Sample	Tool		Cutting Condition			Blade Durability		Surface Roughness
			Cutting Speed	Feed Rate	Cutting Depth	Wear Land Criterion		
			vc, m/min	f, mm/rev	ap, mm	VB, mm	T, min	Ra, μm
AlSi7Mg2Sr003	DCMW	PCD ^1^	500	0.1	0.5	0.16	73.2	1.15
AlSi7Mg2Sr003/SiC 10 vol %	DCMW	PCD ^1^	200	0.15	1.5	0.3	12.3	1.26
	DCMW	PCD ^1^	300	0.1	0.5	0.3	14.7	1.02
	DCMW	PCD ^1^	500	0.1	0.5	0.3	2.5	0.54
	DCMW	PCD ^1^	500	0.2	1.0	0.3	1.7	0.66
	DCMW	PCD ^1^	500	0.3	1.0	0.3	0.5	8.42
	DCMW	PCD ^1^	500	0.1	0.5	0.3	6.3	0.60
	DCMW	PCD ^1^	500	0.3	0.5	0.3	4.1	1.50
	DCGW	PCBN ^2^	100	0.2	0.3	0.3	4.7	5.76
	DCGW	PCBN ^2^	300	0.1	0.5	0.3	0.9	0.80
	DCGW	PCBN ^3^	100	0.2	0.3	0.3	1.2 *	2.56
	DCGW	PCBN ^3^	300	0.1	0.5	0.3	0.7	0.66
	DCGW	PCBN ^4^	100	0.2	0.3	0.3	3.5	6.68
	DCGW	PCBN ^4^	300	0.1	0.5	0.3	1.1	0.55
	CCGX	H10 ^5^	100	0.1	0.5	0.7–1.0	0.8	0.75
	SCGX	H10 ^5^	10	0.1	0.5	0.7–1.0	0.8	1.25
	VCGX	PCD ^6^	300	0.1	0.5	0.3	0.2	0.60

Comments on insert materials: ^1^—MD220 (Mitsubishi); ^2^—MB710 (Mitsubishi); ^3^—MB8025 (Mitsubishi); ^4^—MB4020 (Mitsubishi); ^5^—H10 (Snadvik Coromant); ^6^—PCD sintered at the Institute of Advanced Manufacturing Technology. * Critical wear (blade chipping).

**Table 3:** Comparison of matrix alloy and composite.

Material	Average Mass Loss of 10 Samples *	HB 2.5/61.3
AlSi7Mg2Sr0.03	122.9 mg ± 9.30 mg	80 HB ± 1.4
AlSi7Mg2Sr0.03/SiC_p_ 10 vol %	13.6 mg ± 3.24 mg	99 HB ± 1.7

* After 5000 cycles were carried out with the Taber Rotary Abraser tester (Taber Industries, North Tonawanda, NY, USA) with the abrasion wheels H18, test load of 500 g, suction force of 70% and rotational speed of 60 rev/min.

## References

[1] Degischer H.P., Prader P., Marchi C.S. (2001). “Assessment of metal matrix composites for innovations”—Intermediate report of a European Thematic Network. Compos. Part A Appl. Sci. Manuf..

[2] Koczak M.J., Khatri S.C., Allison J.E., Bader M.G. (2013). Metal-matrix composites for ground vehicle, aerospace, and industrial applications. Fundamentals of Metal-Matrix Composites.

[3] Kainer K.U. (2006). Basics of metal matrix composites. Metal Matrix Composites: Custom-made Materials for Automotive and Aerospace Engineering.

[4] Surappa M.K. (2003). Aluminium matrix composites: Challenges and opportunities. Sadhana.

[5] Ramnath B.V., Elanchezhian C., Atreya T.S.A., Vignesh V. (2014). Aluminum metal matrix composites—A review. Rev. Adv. Mater. Sci..

[6] Chawla N., Chawla K.K. (2006). Metal-matrix composites in ground transportation. JOM.

[7] Hyo L.S., Kyung J.Y., Hee K.Y., Soon H.H. (2000). Fabrication process and thermal properties of SiC_p_/Al metal matrix composites for electronic packaging applications. J. Mater. Sci..

[8] Zhang H., Ye J., Joshi S.P., Schoenung J.M., Chin E.S.C., Gazonas G.A., Ramesh K.T. (2007). Superlightweight nanoengineered aluminum for strength under impact. Adv. Eng. Mater..

[9] Canakci A., Varol T. (2014). Microstructure and Properties of AA7075/Al-SiC composites fabricated using powder metallurgy and hot pressing. Powder Technol..

[10] Ghasali E., Fazili A., Alizadeh M., Shirvanimoghaddam K., Ebadzadeh T. (2017). Evaluation of microstructure and mechanical properties of Al-TiC metal matrix composite prepared by conventional, microwave and spark plasma sintering methods. Materials.

[11] Taha M. (2001). A Practicalization of cast metal matrix composites (MMCCs). Mater. Des..

[12] Beffort O., Long S., Cayron C., Kuebler J., Buffat P.A. (2007). Alloying effects on microstructure and mechanical properties of high volume fraction SiC-particle reinforced Al-MMCs made by squeeze casting infiltration. Compos. Sci. Technol..

[13] Dolata A.J. (2017). Tribological Properties of AlSi12-Al_2_O_3_ Interpenetrating composite layers in comparison with unreinforced matrix alloy. Materials.

[14] Dolata A.J. (2016). Centrifugal infiltration of porous ceramic preforms by the liquid Al alloy—Theoretical background and experimental verification. Arch. Metall. Mater..

[15] Prasad S.V., Asthana R. (2004). Aluminum metal-matrix composites for automotive applications: Tribological considerations. Tribol. Lett..

[16] Tzamtzis S., Barekar N.S., Hari Babu N., Patel J., Dhindaw B.K., Fan Z. (2009). Processing of advanced Al/SiC particulate metal matrix composites under intensive shearing—A novel Rheo-process. Compos. Part A Appl. Sci. Manuf..

[17] Sozhamannan G.G., Prabu S.B., Venkatagalapathy V.S.K. (2012). Effect of processing paramters on metal matrix composites: Stir casting process. J. Surf. Eng. Mater. Adv. Technol..

[18] Prabu S.B., Karunamoorthy L., Kathiresan S., Mohan B. (2006). Influence of stirring speed and stirring time on distribution of particles in cast metal matrix composite. J. Mater. Process. Technol..

[19] Dolata-Grosz A., Dyzia M., Śleziona J., Wieczorek J. (2007). Composites applied for pistons. Arch. Foundry Eng..

[20] Dyzia M. (2011). AlSi7Mg/SiC and Heterophase SiC_P_ + C_G_ composite for use in cylinder-piston system of air compressor. Solid State Phenom..

[21] Dolata A.J., Dyzia M. (2012). Aspects of fabrication aluminium matrix heterophase composites by suspension method. IOP Conf. Ser. Mater. Sci. Eng..

[22] Dolata A.J., Dyzia M., Jaworska L., Putyra P. (2016). Cast hybrid composites designated for air compressor pistons. Arch. Metall. Mater..

[23] Rao R.N., Das S., Mondal D.P., Dixit G. (2010). Effect of heat treatment on the sliding wear behaviour of aluminium alloy (Al-Zn-Mg) hard particle composite. Tribol. Int..

[24] Akbari M.K., Shirvanimoghaddam K., Hai Z., Zhuiykov S., Khayyam H. (2017). Al-TiB_2_ micro/nanocomposites: Particle capture investigations, strengthening mechanisms and mathematical modelling of mechanical properties. Mater. Sci. Eng. A.

[25] Zhiqiang S., Di Z., Guobin L. (2005). Evaluation of dry sliding wear behavior of silicon particles reinforced aluminum matrix composites. Mater. Des..

[26] Singh J., Chauhan A. (2016). Overview of wear performance of aluminium matrix composites reinforced with ceramic materials under the influence of controllable variables. Ceram. Int..

[27] Zhang S., Wang F. (2007). Comparison of friction and wear performances of brake material dry sliding against two aluminum matrix composites reinforced with different SiC particles. J. Mater. Process. Technol..

[28] Viala J.C., Bosselet F., Laurent V., Lepetitcorps Y. (1993). Mechanism and kinetics of the chemical interaction between liquid aluminium and silicon-carbide single crystals. J. Mater. Sci..

[29] Pech-Canul M.I., Ahmad Z. (2011). Aluminum alloys for Al/SiC composites. Recent Trends in Processing and Degradation of Aluminium Alloys.

[30] Ravi K.R., Pillai R.M., Pai B.C., Chakraborty M. (2007). Influence of interfacial reaction on the fluidity of A356 Al-SiC_p_ composites—A theoretical approach. Metall. Mater. Trans. A Phys. Metall. Mater. Sci..

[31] Maleque M.A., Dyuti S., Rahman M.M. Material Selection Method in Design of Automotive Brake Disc. Proceedings of the World Congress on Engineering 2010.

[32] Bigaj M., Boczkal S., Gawlik M., Lech-Grega M. (2015). Analysis of reinforcing particles distribution in composite die-cast pistons. Proceedings of the 14th International Congress for Stereology and Image Analysis.

[33] Boczkal S., Lech-Grega M., Bigaj M., Szymański M. (2015). Final Raport from the Project No. PBS1/B6/13/2012 Financed from Funds Allocated for the National Centre for Research and Development.

[34] Boczkal S., Dolata A.J., Nowak M. (2016). Effect of SiC and GR reinforcement particles on the structure and functional properties of composite casting. Arch. Metall. Mater..

[35] Timpel M., Wanderka N., Schlesiger R., Yamamoto T., Lazarev N., Isheim D., Schmitz G., Matsumura S., Banhart J. (2012). The role of strontium in modifying aluminium-silicon alloys. Acta Mater..

[36] Gautam Krishnan R., Somasekharan Nair E.M. (2013). Study of strontium modification in aluminium alloy. Int. J. Emerg. Technol. Adv. Eng..

[37] Haque M.M. (1995). Effects of strontium on the structure and properties of aluminium-silicon alloys. J. Mater. Process. Technol..

[38] Keith D. (1999). Effects of Magnesium, Silicon and Strontium on the Oxidation of Molten Aluminum. Master’s Thesis.

[39] Pai B.C., Ramani G., Pillai R.M., Satyanaryana K.G. (1995). Review Role of magnesium in cast aluminiurn alloy matrix composites. J. Mater. Sci..

[40] Orlowicz A.W., Mróz M., Tupaj M., Betlej J., Ploszaj F. (2009). Influence of refining process on the porosity of high pressure die casting alloy Al-Si. Arch. Foundry Eng..

[41] Dolata A.J., Dyzia M., Boczkal S. (2016). Influence of the Sr and Mg alloying additions on the bonding between matrix and reinforcing particles in the AlSi7Mg/SiC-C_g_ hybrid composite. Arch. Metall. Mater..

[42] Dolata A.J., Dyzia M., Boczkal S. (2016). Structure of Interface between Matrix Alloy and Reinforcement Particles in Al/SiC_p_ + C_gp_ Hybrid Composites. Mater. Today Proc..

[43] Jaworska L., Putyra P., Czechowski K., Podsiadło M. (2015). Final Raport from the Project No. PBS1/B6/13/2012 Financed from Funds Allocated for the National Centre for Research and Development.

